# Testing the potential of zebularine to induce heritable changes in crop growth and development

**DOI:** 10.1007/s00122-024-04799-3

**Published:** 2025-01-10

**Authors:** E. Jean Finnegan, Peter A. Crisp, Peng Zhang, Judith Eglitis-Sexton, Julian Greenwood, Jessica Hintzsche, Jianbo Li, Jen Taylor, Xiaomei Wallace, Stephen Swain

**Affiliations:** 1 CSIRO Agriculture and Food, Canberra, ACT 2601 Australia; 2 Research School of Biology, Australian National University, Canberra, ACT Australia; 3 School of Agriculture and Food Sustainability, University of Queensland, St Lucia, QLD Australia; 4 Plant Breeding Institute, School of Life and Environmental Sciences, University of Sydney, Cobbitty, NSW 2570 Australia; 5 Queensland Alliance for Agriculture and Food Innovation, University of Queensland, St Lucia, QLD Australia

## Abstract

**Key message:**

**Zebularine-treated wheat uncovered a phenotype with characteristics of an epigenetically regulated trait, but major chromosomal aberrations, not DNA methylation changes, are the cause, making zebularine unsuitable for epigenetic breeding.**

**Abstract:**

Breeding to identify disease-resistant and climate-tolerant high-yielding wheats has led to yield increases over many years, but new hardy, higher yielding varieties are still needed to improve food security in the face of climate change. Traditional breeding to develop new cultivars of wheat is a lengthy process taking more than seven years from the initial cross to cultivar release. The speed of breeding can be enhanced by using modern technologies including high-throughput phenomics, genomic selection, and directed mutation via CRISPR. Here we test the concept of modifying gene regulation by transiently disrupting DNA methylation with the methyltransferase inhibitor, zebularine (Zeb), as a means to uncover novel phenotypes in an elite cultivar to facilitate breeding for epigenetically controlled traits. The development and architecture of the wheat inflorescence, including spikelet density, are an important component of yield, and both grain size and number have been extensively modified during domestication and breeding of wheat cultivars. We identified several Zeb-treated plants with a dominant mutation that increased spikelet density compared to the untreated controls. Our analysis showed that in addition to causing loss of DNA methylation, Zeb treatment resulted in major chromosomal abnormalities, including trisomy and the formation of a novel telocentric chromosome. We provide evidence that increased copy number of the domestication gene,* Q*, is the most likely cause of increased spikelet density in two Zeb-treated plants. Collateral damage to chromosomes in Zeb-treated plants suggests that this is not a viable approach to epigenetic breeding.

**Supplementary Information:**

The online version contains supplementary material available at 10.1007/s00122-024-04799-3.

## Introduction

Wheat is an important dietary staple for much of the world’s population providing about 20% of the calories and protein consumed globally (FAO 2017). Wheat yields have been increased through breeding efforts over many years, but with the dual challenges of an increasingly hostile environment due to climate change and an ever-increasing population there is still a need for new hardy, high-yielding varieties to ensure food security. The development and architecture of the wheat inflorescence are an important component of yield, and both grain size and number have been extensively modified during domestication and breeding of wheat cultivars (Kuchel et al. 2007; Kilian et al. 2010; Peng et al. 2011).

Traditional breeding involves making crosses then selecting F_1_ progeny with desirable traits, followed by successive rounds of inbreeding and selection to produce cultivars that are improvements on those already available. It can take at least seven years to develop a new wheat cultivar, the seed of which then needs to be increased for commercial release (Baenziger 2016). New approaches such as high-throughput phenotyping, the use of molecular markers and genomic selection can improve breeding efficiency, and genome editing may facilitate breeding efforts by introducing targeted genetic changes without the need to perform crosses. Unlike traditional breeding, which selects for phenotypic traits conditioned by all forms of heritable variation, modern breeding techniques based on genomic selection or genome editing are associated with phenotypic variation based on DNA sequence (genetic changes). This is a potential limitation of these approaches as there is growing evidence that heritable phenotypic variation can arise through either genetic and/or epigenetic variation (Springer and Schmitz 2017 and references therein). The concept of using epigenetic variants for crop improvement is currently receiving a lot of interest (Springer and Schmitz 2017; Kawakatsu and Ecker 2019; Varotto et al. 2020; Kakoulidou et al. 2021; Yang et al. 2023).

Epigenetic variation can lead to changes in phenotypes that arise through changes in gene expression determined by chemical modifications of DNA (cytosine methylation) or histone proteins within the associated chromatin that together make up the epigenome. In plants, methylcytosine occurs in different sequence contexts: CG, CHG, and CHH (where H is adenine, cytosine, or thymine); addition of the methyl-group occurs after DNA synthesis and is catalysed by three different classes of DNA methyltransferase. Any effect of DNA methylation on gene expression depends on the location with respect to the promoter and coding region of a gene. Methylation around the transcription start is usually associated with gene silencing (Zhang et al. 2006), and dense methylation of transposable elements and other repetitive sequences maintains them in a quiescent state (Miura et al. 2001; Singer et al. 2001; Lippman et al. 2004). By contrast, methylation within the coding region (gene-body methylation) appears to have little effect on gene expression, particularly when this occurs only in a CG context (Zhang et al. 2006; Bewick and Schmitz 2015). Gene-body methylation is often associated with house-keeping genes that are expressed at moderate levels. While gene-body methylation may not affect expression levels, it can lead to alternate splicing if splice-acceptor sites are modified (Regulski et al. 2013).

There is considerable interest in determining the role of epigenetic variation, with or without associated genetic variation, in regulating phenotypes in both natural populations and crop plants (Eichten et al. 2013; Schmitz et al. 2013; Dubin et al. 2015; Gardiner et al. 2018; Xu et al. 2019; Galanti et al. 2022). Genome-wide methylation analyses have shown that there is considerable variation in DNA methylation patterns between naturally occurring Arabidopsis accessions suggesting that this may contribute to phenotypic variation (Schmitz et al. 2013; Dubin et al. 2015). A similar survey of diverse landraces of bread wheats in the Watkins collection showed that both genotype and DNA methylation profiles were affected by geographic origin although the clustering by DNA methylation occurred across a smaller geographic location, suggesting that it may be more strongly influenced by environment (Gardiner et al. 2018). Consistent with this, changes in DNA methylation have been observed in wheat plants in response to various abiotic and biotic stresses (Kong et al. 2020). Other studies have focussed on epigenetic variation in response to environmental conditions seen in non-crop species (Rajpal et al. 2022 and references therein).

There is evidence that some agronomically important traits can be affected by DNA methylation, for example, dwarfing and disease resistance in rice (Miura et al. 2009; Deng et al. 2017), the mantled phenotype in oil palm (Ong-Abdullah et al. 2015), ripening of tomatoes (Manning et al. 2006) and skin colour in apples (Wang et al. 2020). In addition, DNA methylation plays a role in diploidization of polyploid genomes (Liu et al. 1998; Comai et al. 2000; Shaked et al. 2001; Wang et al. 2004; Zhao et al. 2011; Gardiner et al. 2015; Zhang et al. 2015), suggesting that disruption of DNA methylation could uncover novel phenotypes through the re-activation of silenced genes in polyploid crops such as Brassica, strawberries, cotton, and tetraploid or hexaploid wheats.

Manipulation of DNA methylation has uncovered phenotypic variation in model species as exemplified through the characterization of Epigenetic Recombinant Inbred Lines (EpiRILs) in Arabidopsis (Johannes et al 2009; Reinders et al. 2009; Cortijo et al. 2014). These EpiRILs were generated by crossing two plants that were essentially genetically identical, but which differed in the extent of DNA methylation, due to a mutation in either *methyltransferase I* (*METI*; Finnegan et al. 1993; Reinders et al. 2009) or *decreased DNA methylation 1* (*DDM1*; Kakutani et al. 1995; Johannes et al. 2009), genes required for maintenance of DNA methylation. A collection of EpiRILs was obtained by self-pollination of F_2_ progeny, which lacked the mutation, through multiple generations. The EpiRILs displayed variation in flowering time, plant height, root length, and responses to both biotic and abiotic stresses (Johannes et al. 2009; Reinders et al. 2009; Cortijo et al. 2014).

A similar approach to generating EpiRILs may not be feasible in cereal crops because *MET1* is an essential gene in rice (Hu et al. 2014) and previous efforts to obtain loss of function alleles in maize were not successful (Li et al. 2014). It may be possible to knock out some *MET1* homeoalleles in polyploid wheat without compromising viability. However, treatment with chemicals that inhibit DNA methyltransferase activity, such as 5-Azacytidine (5AzaC) and Zebularine (Zeb), offers an alternative approach to disrupt DNA methylation. Both 5AzaC and Zeb can lead to a substantial reduction in DNA methylation, particularly for CHG and CHH (Baubec et al. 2009; Griffith et al. 2016), which are important in gene silencing in Arabidopsis (Zhang et al. 2006). 5AzaC treatment of *Brassica rapa* gave rise to a population of stochastically hypomethylated plants, some of which showed variation in seed yield and composition (Amoah et al. 2012). Similarly, 5AzaC treatment of strawberry seeds was associated with heritable changes in flowering time and stolon formation (Xu et al. 2016a, b), whereas Zeb treatment of persimmons can affect sex determination (Akagi et al. 2016). We have previously shown that Zeb treatment of young wheat seedlings resulted in a transient reduction in DNA methylation and the appearance of novel phenotypes (Finnegan et al. 2018, 2019).

In this study, we investigated whether transient demethylation could be used as a tool to uncover novel epigenetic traits in an already improved cultivar. To establish proof of concept, we selected a visual, thus easily scored, phenotype that affected the spike architecture in some Zeb-treated wheat plants. The architecture of the spike, including spike density, which is defined as the ratio of spikelets per spike compared to spike length, is one of the determinants of wheat yield (Kuzay et al. 2019). Four genes have been shown to increase spike compactness/density: the major domestication gene, *Q* (*APETALA 2-LIKE 5*, *AP2L5*, chromosome arm 5AL), different homeoalleles of *AP2L2* (chromosome 2), *Compactum* (*C*; chromosome arm 2DL), and *Sphaerococcum* (*S1*, chromosome arm 3DS) (Simons et al. 2006; Xu et al. 2018; Debernardi et al. 2019; Cheng et al. 2020; Wen et al. 2022). The *Rht* dwarfing genes that contributed to increased wheat yields in the green revolution also play a role in determining spike density (Zhao et al. 2018). Several plants with increased spike density were identified in the population of Zeb-treated plants and were analysed to determine the heritability and stability of this visual trait over multiple generations. A molecular analysis of these plants showed that the altered phenotype was associated with increased expression of *Ap2-like* transcription factors, but this was not caused by an altered pattern of DNA methylation.

## Materials and methods

### Zebularine treatment

Five batches of seed (1,000 seed per batch) of a glasshouse grown semi-spring cultivar, Sunstate (SS) (Eagles et al. 2009), were treated with 400 µM Zebularine according to Finnegan et al. (2018) and then transferred to soil. One hundred mock-treated seedlings were grown in parallel as controls for each of the five batches. Treated plants were scored for altered phenotypes under glasshouse conditions, 18⁰C day/13⁰C night with natural light supplemented by metal arc lights to achieve photoperiods of 12-h light (April—September) or 16-h light (October—March). Plants (test and control) were sown in soil 50-mm square pots and seeds were harvested from individual plants, but where a plant had more than one fertile spike, the seed from all spikes was pooled. Plants from later generations were grown in 10-cm round pots.

### Nomenclature

Plants grown from Zeb-treated seedlings were designated as eM_1_ followed by a number from Z1 to Z1685, the number of plants that survived the treatment. The self-pollinated progenies of treated plants were designated as eM_2_ Zn.1, Zn.2, and so on; the progenies of eM_2_ Zn.1 were named eM_3_ Zn.1.1, Zn.1.2, and so on. The naming of plants in each generation followed this pattern so the pedigree of each plant can be readily determined.

### Scoring the clubhead (CH) phenotype

Scoring of the CH phenotype was initially done by a visual inspection, but in some instances, when the CH phenotype was mild (i.e. affected very few internodes and/or the spike resembled those on SS controls) the length of internodes in the spike was measured using a pair of digital callipers. The top internode of SS controls (immediately below the terminal spikelet) was 5.15 ± 0.02 mm (n = 568). Plants were scored as having a CH phenotype when the top internode was < 4 mm. Plant height was measured from the soil to the top of the spike on the longest tiller with a steel metre rule. Stem length was measured from the soil to the base of the spike. Spike length was measured from the base of the first spikelet (partially or fully developed) to the tip of the terminal spikelet, excluding the awns.

### RNA isolation and cDNA synthesis

Total RNA extractions were carried out using the Maxwell® RSC Plant RNA Kit (Promega Corporation), according to the manufacturer’s protocol. RNA concentration was measured with a Nanodrop 2000 (Thermo Fisher Scientific Inc.), and 15 µg of each sample transferred and dried onto GenTegra-RNA™ tubes (GenTegra LLC) until required for further analysis. RNA samples were diluted to 1 µg per 10 µL for cDNA synthesis using UltraPure water, and first-strand cDNA synthesis was performed with Superscript III Reverse Transcriptase (Invitrogen) using an oligo(dT)18VN primer at 2.5 µM final concentration and a dNTP mix at a final concentration of 1 mM in a 20 µl reaction.

### Quantitative real-time PCR

RT-qPCR was conducted to measure* Q* gene (transcript ID XM_044527740.1) expression using the reference gene glyceraldehyde 3-phosphate dehydrogenase (GAPDH, transcript ID XM_044563950.1). The Primer3 software was used to design the following primers: Q-forward GAAGTTGAAGCTGCAAGGGCG, and Q-reverse, ATTGCCTCATTTTCGGCGTCGG, GAPDH-forward AACGAGTGGGGATACAGCACCC and GAPDH-reverse CAAACCACTCTCCCCTGTATGCC. The Promega GoTaq® 1-Step RT-qPCR System was followed as per the manufacturer’s instructions, using the GoTaq® qPCR Master Mix. The 20 µL cDNA product was diluted with 180 µL of UltraPure water and 4.5 µL of cDNA used per qPCR reaction with 5.5 µL of master mix containing primers, and each biological replicate was measured in technical triplicate. Gene expression was compared between samples using the delta-delta Ct method.

RT-qPCR to measure the expression of *Ap2L-A2* and *Ap2L-B2* was carried out as described in Finnegan et al. (2018); primer sequences are available in Debernardi et al. (2020).

### Genomic sequencing

Genome re-sequencing to analyse chromosomal aberrations and changes in copy number was undertaken by preparing DNA sequencing libraries using the Illumina Nextera XT DNA Library Preparation Kit as per the manufacturer instructions. Genomic DNA was extracted from leaf tissue using the Maxwell® RSC PureFood GMO and Authentication Kit as per the manufacturer’s instructions with the addition of the optional RNase treatment. One nanogram DNA was used as input for library preparation. Libraries were sequenced on an Illumina Novaseq X S4 lane in 150 PE mode at Australian Genomics Research Facility (Supplementary Table 1).

### Fluorescence in situ hybridization (FISH) analysis

Mitotic metaphase chromosome preparations from root tips of lines Z11 and Z1021, and cv. Sunstate followed Lang et al. (2018). FISH was performed as described in Li et al. (2020). Oligo probes Oligo-pSc119.2 and Oligo-pTa535 were 5′-end labelled with 6-carboxyfluorescein (6-FAM) and 6-carboxytetramethylrhodamine (Tamra) (Merck, Bayswater, Australia) producing green and red signals, respectively. The unique hybridization patterns they produced enabled identification of individual chromosomes. Chromosomes were counterstained with 4′,6-diamidino-2-phenylindole (DAPI) (Invitrogen Life Science, Carlsbad, CA, USA) and pseudo-coloured blue.

## Results

### Phenotypic variation observed in the eM_1_ generation

**Fig. 1:**
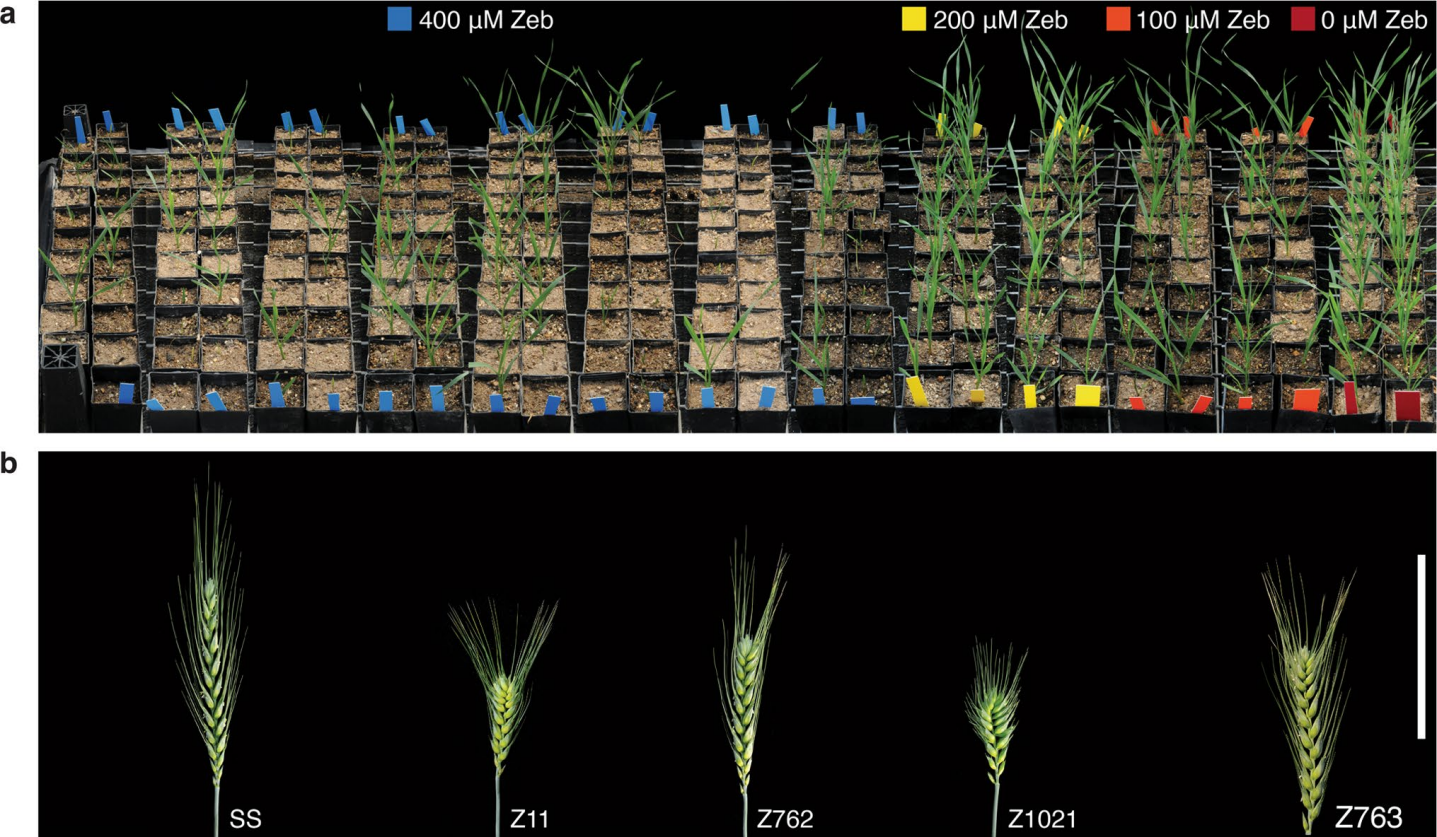
Treatment with zebularine affects plant growth and development. **a** Treated plants were transferred to soil and allowed to develop to maturity. Plants in this study were treated with 400 µM which kills approximately 70% of seedlings. Even plants treated with lower concentrations of Zeb (100 µM or 200 µM) show reduced vigour and viability. **b** CH spikes from each of the four families Z11, Z762, Z1021, and Z763. Scale bar = 100 mm

Loss of DNA methylation during Zeb treatment could activate gene expression giving rise to a gain-of-function, dominant trait (Cheng et al. 2004; Baubec et al. 2009). We have previously reported that exposure of young wheat seedlings to 400 µM Zebularine (Zeb) for three days leads to 16–28% reduction in the level of 5-methylcytosine (5-mC), as estimated by HPLC. The level of 5-mC recovered to wildtype level one month after the treatment, raising the question of whether any sites were stably demethylated and inherited by the progeny of treated plants (Finnegan et al. 2018, 2019). It is likely that demethylated sites that are not subject to RNA-directed DNA methylation will remain unmethylated in progeny (Johannes et al. 2009). Plants treated with Zeb (eM_1_ generation) generally showed reduced vigour and were both shorter in stature and less fertile than the non-treated controls (Fig. 1a), most likely due to the toxic effects of Zeb (Baubec et al. 2009; Cho et al. 2011).

Eight (0.6%) eM_1_ plants had an altered spike architecture with shortened rachis internodes at the top of the spike, a phenotype that we termed clubhead (CH; Fig. 1b). As the meristems that give rise to the first few tillers are present in the embryo prior to germination, any demethylation following Zeb treatment would be expected to differ between individual tillers on the same plant. The clubhead phenotype was restricted to one spike on eM_1_ plants that had multiple tillers.

### Phenotyping of the eM_2_ generation

To distinguish between phenotypes that were due to Zeb toxicity and those that had a molecular (genetic or epigenetic) basis, 12 seeds from each of 235 eM_1_ plants from this population, including all eM_1_ plants showing the clubhead phenotype, were planted in soil and screened for novel phenotypes in the eM_2_ generation. Several other novel phenotypes were observed in plants of the eM_2_ generation including delayed flowering, altered plant height and enhanced or reduced dormancy. Changes in dormancy could not have been observed in the eM_1_ generation, but altered flowering time and plant height may not have been observed in treated plants because of the adverse effect of Zeb treatment. Of these, both dormancy phenotypes were transmitted to the eM_3_ generation (data not shown); the late flowering phenotype, which was also heritable, has been described elsewhere (Finnegan et al. 2018).

The remainder of this study focussed on the clubhead (CH) phenotype, which appeared to be a dominant trait as it was observed in Zeb-treated (eM_1_) plants. This phenotype was transmitted to eM_2_ progeny in six out of eight lines (Table 1). Two families, Z307 and Z1168, both of which showed a low frequency of transmission to the eM_2_ progeny (29% and 28%, respectively), were not investigated further based on our experience with poorly inherited late flowering phenotypes that disappeared in subsequent generations (Finnegan et al. 2018).

**Table 1:** Segregation of clubhead (CH) and normal (N) phenotype in the eM_2_ progeny of treated eM_1_ plants that had a CH spike

Plant family	Frequency of CH progeny in eM_2_ (%)	Number scored	χ^2^_3:1_
Z11	56	25*	5.412
Z307	29	17**	20.923
Z762	100	8^a^	
Z763	100	5^a^	
Z1021	46.7	15*	5.455
Z1168	28	36**	42.815
Z1438	0	50	
Z1442	0	26	

^a^100% progeny in eM_3_ and subsequent generations of each of these plants displayed the CH phenotype

^*^p < 0.05; **p < 0.00001. The probability has been assessed in a χ^2^ test based on an expected 3 (CH): 1 (N) segregation

### The clubhead phenotype is dominant and affects spike density, spikelet development and plant height

To verify that the CH phenotype was conditioned by a dominant (epi)-mutation in the remaining four families, plants with the CH phenotype were backcrossed to non-treated Sunstate (SS) plants. For each of these families (Z11, Z762, Z763 and Z1021), some F_1_ progeny displayed the CH phenotype, although the frequency of transmission varied between families. Transmission of the CH phenotype to F_1_ progeny in all four families confirms the dominant nature of the CH trait, which was implicated by appearance of the CH phenotype in eM_1_ plants.

**Fig. 2:**
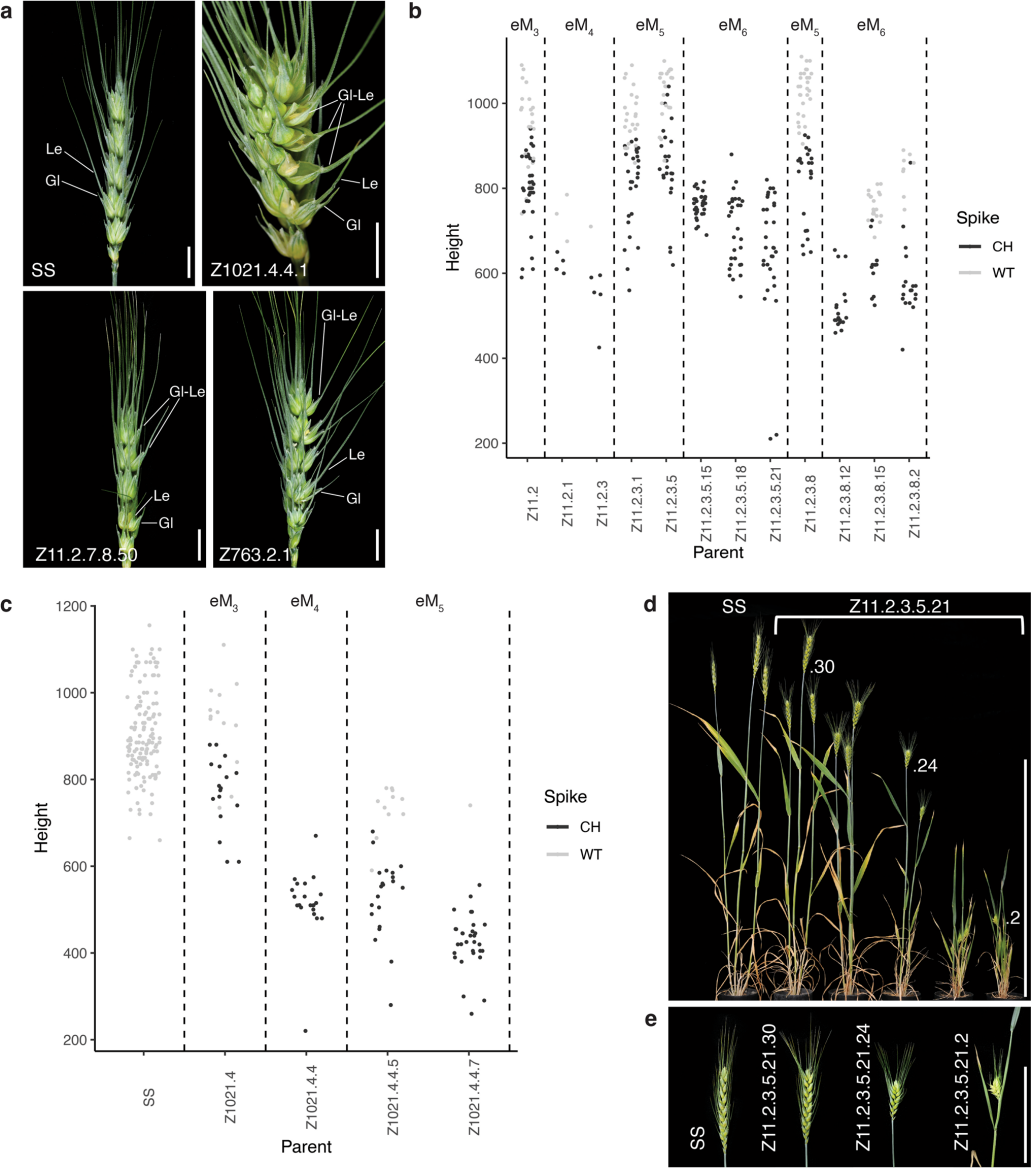
Severity of the CH phenotype, which affects not only spike architecture but also plant height and spikelet development, is variable among sibling plants. **a** Spikelet development is altered in CH spikes. The sterile glumes are replaced by organs that resemble a glume/lemma hybrid. Gl = glume; Le = lemma; Gl-Le – Glume/lemma which have an elongated awn (compared to the awn on a glume) and adaxial to which is a rudimentary or fully fertile floret (Greenwood et al. 2017). Scale bars = 10 mm. **b** Scatter plots showing the heights of siblings from four generations of plants from family Z11. In general, plants with normal spike architecture were taller than CH siblings. The corresponding scatter plot for non-treated SS is shown in (d). The height of plants depended to some extent on the season as plants were grown in a polytunnel with daylight extension from April to September. This is reflected in the range of plant height for SS controls. **c** Scatter plots showing the heights of siblings from three generations of plants from family Z1021. The height of plants depended to some extent on the season as plants were grown in a polytunnel with daylight extension from April to September. This is reflected in the range of plant height for SS controls. **d** The severity of the CH phenotype varies in the progeny of homozygous plant eM_5_ Z11.2.3.5.21, ranging for a mild CH to an extreme dwarf with sterile spike. Scale bar = 500 mm. **e** Spikes from three of the sibling plants shown in (b). Scale bar = 100 mm

We observed several other changes to spike architecture and development in plants showing the CH phenotype. Firstly, spike density (number of spikelets/length of the spike) significantly increased in CH spikes (3.3 ± 0.05 spikelets/cm ± SEM) compared to heads with normal architecture (2.4 ± 0.02; Supplementary Table 2). The development of spikelets, which normally comprise two basal, sterile glumes subtending an indeterminate number of florets was also affected by the CH (epi)-mutation. In CH spikes, glumes were sometimes replaced by rudimentary florets, lacking some floral organs, or by complete florets adaxial to glume/lemma like organs with elongated awns (Fig. 2a). Plants with CH spikes were generally shorter in stature than sibling plants with normal spikes. In CH plants, all the internodes of the stem were shortened compared to their normal siblings with the top two internodes being more severely affected (Table 2).

**Table 2:** Lengths of stems and internode in plants with clubhead (CH) spikes and normal (N) siblings^a^

Phenotype	Stem length^a^ (mm)	Internode^b^ 1	Internode^b^ 2	Internode^b^ 3	Internode^b^ 4
N	674.6 ± 9.2	347.8 ± 5	163.4 ± 2.6	103.3 ± 2.2	60.1 ± 3.2
CH	464.3 ± 32.8 ****	210 ± 16.6 ****	122.3 ± 7.5 ***	76.5 ± 1 5.6 **	45.1 ± 4.1*

^a^The stem length (excluding the spike) of plants was measured for sibling progeny of plant eM_5_ Z1021.4.4.5 (18 plants with N spike; 21 plants with CH spike)

^**b**^Internode 1 is the internode immediately below the spike; Internode 2 is below Internode 1, etc.

Significance was tested by a two-tailed T test with unequal variance **** P < 0.0001; *** P < 0.005; ** P < 0.001; * P < 0.05

### Variable transmission in subsequent generations

Zeb is a competitive inhibitor of DNA methyltransferases that causes demethylation at random sites throughout the genome. We therefore anticipated that the Zeb-induced change that conditions the CH phenotype would exist in a heterozygous state in the eM_1_ generation. Surprisingly, the treated plants (eM_1_) in families Z762 and Z763 appeared to be homozygous for the causal molecular change because all progeny in the eM_2_ and subsequent generations inherited the CH phenotype (Table 1). Consistent with this, all F_1_ plants from reciprocal crosses between Z762 and SS displayed the CH phenotype. Although not all F_1_ progeny of crosses involving Z763 showed the CH phenotype, this phenotype segregated in F_2_ progeny derived by self-pollinating F_1_ plants with normal spike architecture, suggesting that penetrance of CH in heterozygous plants was not always 100%. Penetrance of the CH phenotype may be dependent on the level of gene expression, which would be lower in the heterozygous state.

The transmission frequency of the CH phenotype to self-pollinated progeny in the eM_2_ generation does not fit with the expected 3 CH:1 normal (N) segregation for a dominant Mendelian trait, for the remaining two families. For family Z11, 56% of progeny showed the CH phenotype (χ^2^ = 5.412, p < 0.05) while for family Z1021 47% of progeny were CH (χ^2^ = 5.455, p < 0.05) (Table 1). There was some variability in the severity of the CH phenotype in the self-pollinated progeny of individual plants in these two families (Fig. 2b, c). We considered the possibility that the severity of the phenotype, which in some plants was associated with dwarfing as well as an increased number of shortened internodes in the spike (Fig. b, c), could relate to the zygosity of the causal (epi)-mutation that conditions the CH spike. Consistent with this, the CH phenotype in families Z762 and Z763, which appear to be homozygous, showed very little variation in severity.

We therefore sought plants that were true breeding as evidence for homozygosity for the (epi)-mutation in families Z11 and Z1021, by identifying plants that had 100% progeny displaying the CH phenotype. Several homozygous plants were obtained for both Z11 and Z1021 after five and two generations of self-pollination, respectively (Supplementary Fig. S1). In the process of identifying homozygous plants, we noted that the pattern of inheritance of the CH phenotype was significantly different from the 3:1 segregation expected for a dominant Mendelian trait as had been observed in the eM_2_ progeny populations (Supplementary Fig. S1). These observations suggest that the CH phenotype may not be conditioned by a simple genetic trait and could be associated with demethylation/remethylation of site(s) regulating expression of a gene conditioning the CH phenotype. Alternatively, under-representation of plants with the CH phenotype would be consistent with incomplete penetrance as noted for Z763, or with poor transmission of the chromosome conditioning the phenotype as Zeb treatment can cause chromosomal aberrations (Cho et al. 2011; Ma et al. 2016).

### The CH phenotype was lost in some progeny of plants identified as homozygous

**Fig. 3:**
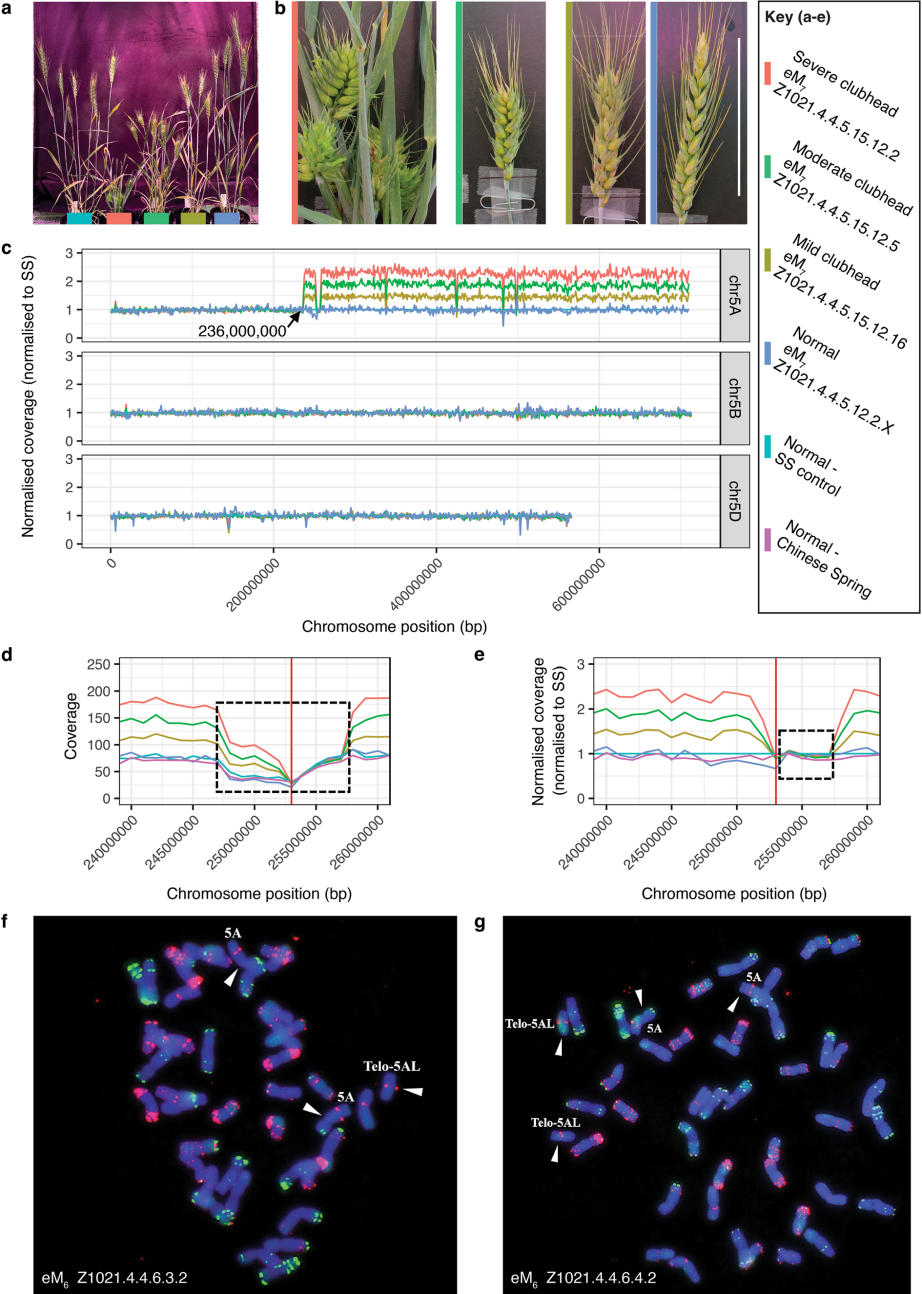
CH phenotype in family Z1021 is associated with the presence of a novel telocentric chromosome 5A (t5AL) **a** The severity of the CH phenotype varies among sibling progeny of homozygous plant Z1021.4.4.5.15.12. **b** Spikes from the individual plants from left to right: Z1021.4.4.5.15.12.2, Z1021.4.4.5.15.12.16, Z1021.4.4.5.15.12.5 and Z1021.4.4.5.12.2.16, shown in (a). Scale bar = 100 mm. **c** Genome sequence traces showing an increase in copy number of the long arm of Chromosome 5A in DNA from plants shown in (a) & (b). Z1021.4.4.5.15.12.2 (5 copies of 5AL), Z1021.4.4.5.15.12.16 (4 copies of 5AL), Z1021.4.4.5.15.12.5 (3 copies (of 5AL)), and a pool of three plants Z1021.4.4.5.12.2.X including .16 (2 copies of 5A), compared to SS. **d** Sequence coverage of the centromeric region for chromosome t5AL in plants. The region spanning bases 247,000,000—259,000,000 (dashed box) shows reduced coverage in SS relative to CS. Red line marks the estimated start of the centromere in Chinese Spring. **e** Sequence coverage of the centromeric region for chromosome t5AL normalized to SS. The region from 252,600,000 bp to 258,300,000 bp is not increased in copy number compared to SS. **f** FISH analysis of Z1021.4.4.6.3.2 which has one copy of t5AL in addition to two normal 5A chromosomes. **g** FISH analysis of Z1020.4.4.6.4.2 which has two copies of t5AL in addition to two normal 5A chromosomes

We scored the self-pollinated progeny of plants identified as homozygous to determine whether the CH phenotype was more uniform and stably inherited. We were surprised to observe a gradation of phenotypes, ranging from mild to very severe, among the progeny of homozygous plants from both Z11 and Z1021 (Fig. 2d; Fig. 3a). Some plants had a weak phenotype where only the top 1–3 internodes of the spike were reduced in length, and the plants were comparable in height to the SS controls. By contrast, severely affected plants were extreme dwarfs, with sterile spikes in which every internode was reduced in length (Fig. 2d, e; Fig. 3a, b). The severe phenotype was observed in only a few plants (6% [2/32] for Z11 and from 4 to 9.5% [1/25 to 2/21] for Z1021) in some progeny populations.

We determined plant eM_3_ Z1021.4.4 to be homozygous because all 32 progeny in the eM_4_ generation displayed the CH phenotype (Fig. 2c). We scored the self-pollinated progeny from 16 of these eM_4_ plants, 12 of which also gave rise to 100% plants with CH phenotype (Table 3). The remaining four plants had a variable number of progenies (3—37%) with normal spike architecture (Fig. 2c; Supplementary Fig. S1b; Table 3). We considered the possibility that eM_3_ Z1021.4.4 was not homozygous for the causal (epi)-mutation. However, we could not reconcile the inferred genotype in the eM_4_ generation, where 12 out of 16 plants appeared to be homozygous for the (epi)-mutation while the phenotype segregated in the remaining four plants, with heterozygosity for a single dominant Mendelian trait (χ^2^ = 22.0, p = 0.0002 for 1:2:1 CHCH:CHN:NN). This raised the question of whether the appearance of progeny with normal spike architecture was associated with incomplete penetrance or whether the molecular change causing the CH phenotype had reverted or had not been transmitted to the progeny with normal spikes.

**Table 3:** Genotyping of eM_4_ self-pollinated progeny of homozygous plant eM_3_ Z1021.4.4

Plant	CH progeny (%)	Number scored^a^
Z1021.4.4.2	100^b^	21
Z1021.4.4.3	100	24
Z1021.4.4.5	63	30^d^
Z1021.4.4.6	100	8
Z1021.4.4.7	97	30^e^
Z1021.4.4.9	100	28
Z1021.4.4.10	100	25
Z1021.4.4.11	100^c^	25
Z1021.4.4.12	100	10
Z1021.4.4.13	100	27
Z1021.4.4.14	100	27
Z1021.4.4.15	94	32
Z1021.4.4.16	78	32
Z1021.4.4.17	100^c^	24
Z1021.4.4.18	100	29
Z1021.4.4.19	100	31

^a^32 seeds were planted for all but lines Z1021.4.4.6, Z1021.4.4.12, and Z1021.4.4.18, but not all seed germinated giving rise to the variation in number of plants scored

^b^2 sterile, extreme dwarfs among progeny

^c^1 sterile extreme dwarf among progeny

^d^3/10 ‘normal’ plants tested had 100% N progeny (Table 4)

^e^1/1 ‘normal’ plant had 100% N progeny

To investigate this further, we scored the spike phenotype of eM_6_ progeny derived by self-pollinating 26 plants in eM_5_ population (Z1021.4.4.5.n) where the CH phenotype was segregating (Table 4). Three eM_5_ plants with normal spikes gave rise to 100% normal progeny, which continued to breed true in subsequent generations. This suggested that the CH phenotype had been lost by reversion or that the causal molecular change had not been transmitted through meiosis. Seven other plants from the eM_5_ generation that had normal spike architecture had a few progenies with CH spikes (3–22%; Table 4), an observation more consistent with incomplete penetrance. Three plants scored as CH in the eM_5_ generation appeared to be homozygous for the CH (epi)-mutation. In the remaining 13 plants, the CH phenotype segregated at ratios ranging from 3 CH:1 N to 1 CH:1 N (Table 4). These observations were repeated for sibling line eM_4_ 1021.4.4.7, where one eM_5_ plant had 100% normal progeny.

**Table 4:** Segregation of clubhead (CH) and normal (N) phenotype in the progeny of eM_4_ Z1021.4.4.5^a^

Plant (eM_5_)	Phenotype	Inferred genotype	CH in eM_6_ progeny (%)	Number scored^b^	χ^2^_3:1_
Z1021.4.4.5.3	CH	Heterozygous	69	16	0.333
Z1021.4.4.5.4	N	?	9***	32	73.5
Z1021.4.4.5.5	N	?	22***	32	46.167
Z1021.4.4.5.6	N	?	3***	32	88.167
Z1021.4.4.5.7	CH	Heterozygous	63	32	2.667
Z1021.4.4.5.8	CH	Heterozygous?	55*	31	6.065
Z1021.4.4.5.9	CH	Heterozygous?	50*	22	5.729
Z1021.4.4.5.10	CH	Heterozygous	71	28	0.19
Z1021.4.4.5.11	CH	Heterozygous?	53**	32	8.167
Z1021.4.4.5.12	N	WT	0	32	96
Z1021.4.4.5.13	CH	Heterozygous?	50**	32	10.667
Z1021.4.4.5.14	CH	Homozygous	100	23	
Z1021.4.4.5.15	CH	Homozygous	100	22	
Z1021.4.4.5.16	CH	Heterozygous	69	32	0.667
Z1021.4.4.5.17	N	WT	0	32	96
Z1021.4.4.5.18	CH	Heterozygous	56	16	3.0
Z1021.4.4.5.19	CH	Heterozygous?	56*	32	6.0
Z1021.4.4.5.21	N	?	9***	32	73.5
Z1021.4.4.5.22	N	?	12***	32	66.667
Z1021.4.4.5.23	N	?	21***	32	48.167
Z1021.4.4.5.24	CH	Heterozygous	69	32	0.667
Z1021.4.4.5.25	N	?	6***	32	80.667
Z1021.4.4.5.26	CH	Heterozygous	75	32	0
Z1021.4.4.5.27	CH	Heterozygous?	53**	32	8.167
Z1021.4.4.5.28	CH	Homozygous	100	21	
Z1021.4.4.5.30	N	WT	0	32	96

^*^p < 0.05; **p < 0.01; ***p < 0.001. The probability has been assessed in a χ^2^ test based on an expected 3 (CH): 1 (N) segregation assuming that the normal plants were mis-scored due to incomplete penetrance. ^a^The genotype of plants has been inferred by phenotyping their progeny. Plants designated as ‘heterozygous’ showed a 3:1 segregation of CH and N phenotype in progeny. Plants where the segregation was significantly different from 3:1 were designated as ‘heterozygous?’ Where plants with N spike architecture produced a few CH progeny, the inferred genotype was designated as ‘?’. ^b^32 seeds were planted for all except lines Z1021.4.4.5.3 and Z1021.4.4.5.18, but not all seed germinated giving rise to the variation in number of plants scored

Incomplete penetrance of the phenotype does not adequately explain the complete absence of CH spikes in the progeny of three eM_5_ plants from this line. Other possibilities for the irregular behaviour of the CH trait in family Z1021 include remethylation of site(s) demethylated following Zeb treatment, the independent segregation of more than one (semi-)dominant gene contributing to the CH phenotype, or the association of the CH phenotype with a chromosomal rearrangement that is not stably transmitted through either male and/or female gametes. The segregation data do not enable us to discriminate between these alternatives. By contrast, the CH phenotype seemed to be more stably inherited from plants in the Z11 family that were identified as homozygotes, as we identified putative homozygotes through four generations of self-pollination (Supplementary Fig. S1a). This suggests that the CH phenotype was more stable in this line than in Z1021, although in later generations the severity of the phenotype decreased and the frequency of incomplete penetrance increased (data not shown).

### The CH phenotype in families Z1021 and Z11 is associated with a partial or complete duplication of chromosome 5A

**Fig. 4:**
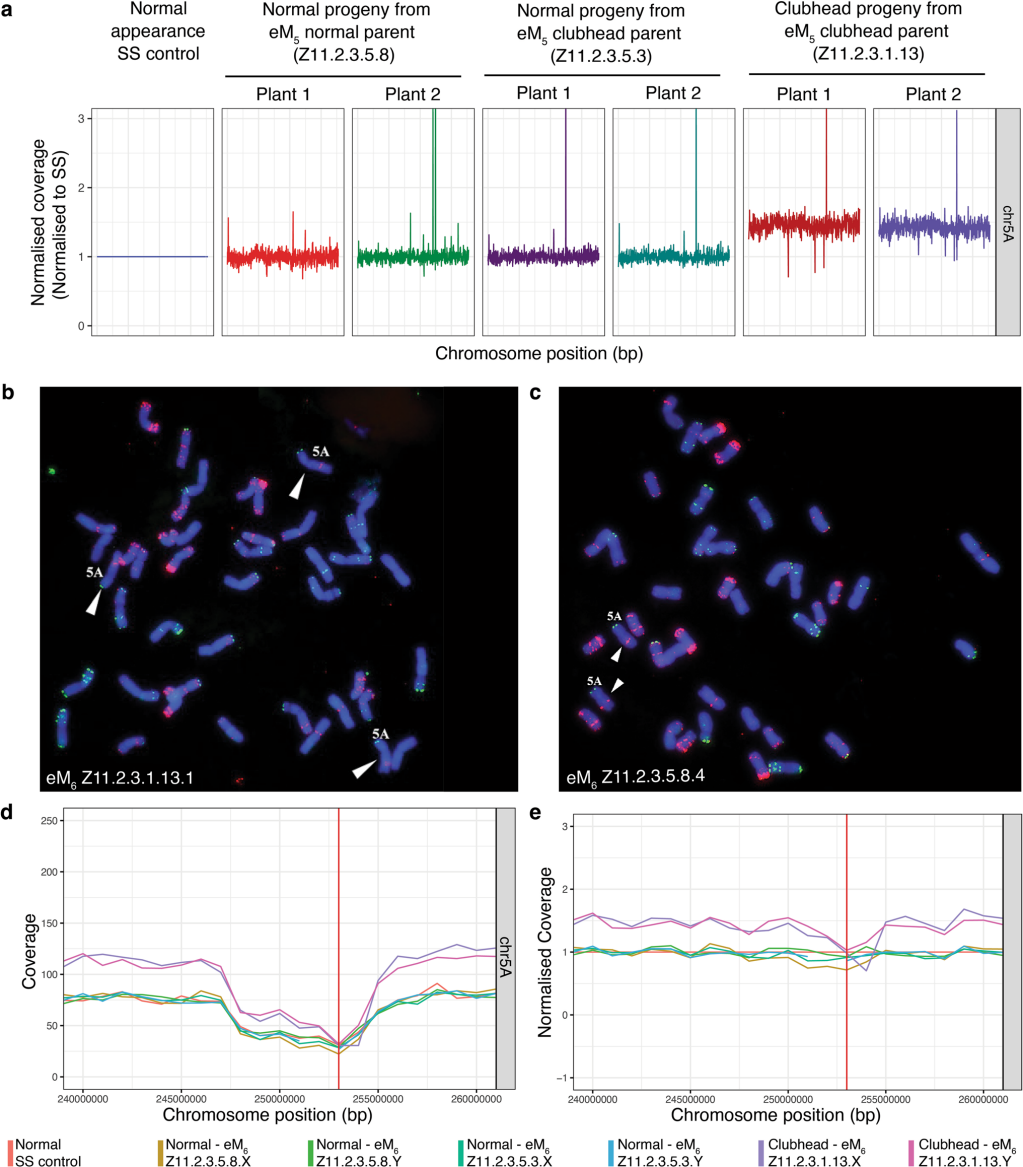
CH phenotype in family Z11 is associated with the presence of an additional copy of chromosome 5A **a** Genome sequence traces for Chromosome 5A in DNA from family Z11 compared to SS. Plants with normal appearance (two progeny each of eM_5_ Z11.2.3.5.8 and Z11.2.3.5.3) have 2 copies of chromosome 5A, whereas plants with CH spike (two progeny of eM_5_ Z11.2.3.1.13) have 3 copies of chromosome 5A. **b** FISH analysis of CH plant Z11.2.3.1.13.1 which has three copies of chromosome 5A. **c** FISH analysis of normal plant Z11.2.3.5.8.4 which has two copies of chromosome 5A. **d** Sequence coverage of the centromeric region for chromosome 5A. The region spanning bases 247,000,000—259,000,000 shows reduced coverage in SS relative to CS. Red line marks the estimated start of the centromere in Chinese Spring. **e** Sequence coverage of the centromeric region for chromosome 5A normalized to SS. The region from ~ 252,500,000 – 254,500,000 bp is not increased in copy number in Z11.2.3.1.13.Y compared to SS. This region may have been deleted from 2 copies of 5A in plant Z11.2.3.1.13.X as coverage is even lower in this plant

To investigate the possibility that there was a chromosomal rearrangement associated with the CH phenotype, we undertook low pass genome re-sequencing. Skim-sequencing, sometimes termed as 'digital karyotyping', is an effective means to identify chromosomal aberrations or changes in DNA copy number (Shrestha et al. 2023). In both families Z11 and Z1021, increases in the copy number of chromosome 5A were identified based on normalized read depth relative to control plants and to the other chromosomes. In family Z1021, we compared the DNA sequence data from a pool of SS controls, a pool of normal looking progeny plants from the reverted eM6 parent (Z1021.4.4.5.12.2) and three individual progeny of an eM_6_ CH parent (Z1021.4.4.5.15.12). The three CH individuals were classified as mild CH, moderate CH, and severe CH (Fig. 3a, b). Each of the CH plants had an increase in copy number for chromosome 5A starting from position ~ 236,000,000 bp and continuing to the end of the chromosome, with a copy number increase of 1.5, 2 and 2.5 for the mild, moderate, and severe phenotype, respectively (Fig. 3c). The other chromosomes were not affected (Fig. 3c). For family Z11, we sequenced DNA isolated from two normal looking progeny from a normal eM_5_ parent (Z11.2.3.5.8), two predominately normal progeny from a CH parent (Z11.2.3.5.3), and two CH progeny from a CH parent (Z11.2.3.1.13). In this case, the progeny with CH appearance had an increased copy number of 1.5 for the whole of chromosome 5A (Fig. 4a). In addition, one of the CH plants also displayed a loss of one copy of 6A (data not shown). Variation in copy number of chromosome 5A (from 2n) is associated with variation in phenotype, such that the higher the copy number (5A plus the duplicated 5A), the more severe the phenotype in plants from family Z1021. This strongly suggests that an increase in copy number of chromosome 5A cause the CH phenotype.

The sequencing of DNA from family Z1021 identified six regions within the duplicated segment of 5A where the copy number was not increased relative to the SS control (Fig. 3c). Closer examination showed that three of these regions corresponded to structural variations within the SS genome where these regions were absent in SS, but present in Chinese Spring (CS) RefSeq v1.0 (International Wheat Genome Sequencing Consortium 2018) (Supplementary Fig. S2a, b). Two regions were most likely associated with artefacts, as coverage of these regions was abnormally high in SS (Supplementary Fig. S2c, d). A stretch of approximately 10.7 Mb (~ 247,000,000 bp to ~ 257,700,000 bp) showed reduced coverage in SS compared to the CS reference sequence (Fig. 3c); this region most likely corresponds to the 5A centromere, based on map locations of the 5A centromere in other genotypes (Walkowiak et al. 2020). It is possible that the reduced coverage of pericentric DNA is a sequencing artefact as the centromeric regions of chromosomes 5B and 5D are also somewhat underrepresented in SS relative to CS (Supplementary Fig. S3b). The remaining segment (~ 252,600,000 bp to ~ 258,300,000 bp) where the copy number was not increased relative to SS lies within the pericentric region. Here the copy number was not increased relative to SS no matter how many copies of the partially duplicated chromosome 5A were present (Fig. 3d, e). This suggests that a 5.7-Mb region has been deleted from the duplicated 5A chromosome.

We also observed that a region of pericentric DNA had been deleted from the additional copy of chromosome 5A present in CH plants from family Z11 (Fig. 4d, e). The deleted segment was somewhat smaller in Z11 (2.0 Mb; ~ 252,500,000 bp to ~ 254,500,000 bp) and lies almost completely within the deletion seen in Z1021.

### FISH analysis shows that Z1021 has a novel telocentric chromosome 5AL

Fluorescence in situ hybridization (FISH) analysis was performed on chromosome spreads of plants descended from the homozygous eM_5_ sibling lines, Z1021.4.4.6.3 and Z1021.4.4.6.4. One of the plants, Z1021.4.4.6.3.1, has one copy of 5A and three copies of a novel telocentric (t) chromosome 5AL (t5AL) (Table 5). The other plant, Z1021.4.4.6.3.2, has two copies of 5A and one copy of t5AL (Fig. 3f). For the other line, plant Z1021.4.4.6.4.1, has the same chromosome composition as Z1021.4.4.6.3.2 (Table 5). The other plant, Z1021.4.4.6.4.2, has two copies of both 5A and t5AL (Fig. 3g). Based on the sequence data, t5AL comprises the complete long arm and centromere with the break point on the short arm being adjacent to the centromere (Fig. 3c). The number of t5AL chromosomes ranged from one to three copies, although unlike the severe CH plant subjected to genome sequencing, the plant with three t5AL had only one copy of the intact chromosome 5A (Table 5).

**Table 5:** Karyotype of plants from eM6 generation of families Z11 and Z1021

Plant	Parent	Chromosome number (2n)	Copy number chromosome 5A^a^
SS	N	42	5A’’
Z11.2.3.5.3.1	CH (heterozygous)	42	5A’’
Z11.2.3.5.3.2	CH (heterozygous)	42	5A”
Z11.2.3.5.8.3	N	42	5A’’
Z11.2.3.5.8.4	N	42	5A’’
Z11.2.3.1.13.1	CH (homozygous)	43	5A’’’
Z1021.4.4.6.3.1	CH (homozygous)	44	5A’ + t5AL’’’
Z1021.4.4.6.3.2	CH (homozygous)	43	5A’’ + t5AL’
Z1021.4.4.6.4.1	CH (homozygous)	43	5A’’ + t5AL’
Z1021.4.4.6.4.2	CH (homozygous)	44	5A’’ + t5AL’’

^a^The symbols’,’’, and’’’ indicate one, two, and three copies, respectively, for chromosomes 5A or t5AL

FISH analysis was also performed on the chromosome spreads of plants from line eM_6_ Z11.2.3.5.3 (two plants), Z11.2.3.5.8 (two plants; Fig. 4c), and Z11.2.3.1.13 (one plant; Fig. 4b). Both plants of Z11.2.3.5.3 and Z11.2.3.5.8 had the normal complement of 42 chromosomes (two copies of chromosome 5A: normal head type), and the plant in Z11.2.3.1.13 had 43 chromosomes with three copies of chromosome 5A (clubhead type) (Table 5).

### *Q* gene expression was elevated in plants of the Z11 and Z1021 families

**Fig. 5:**
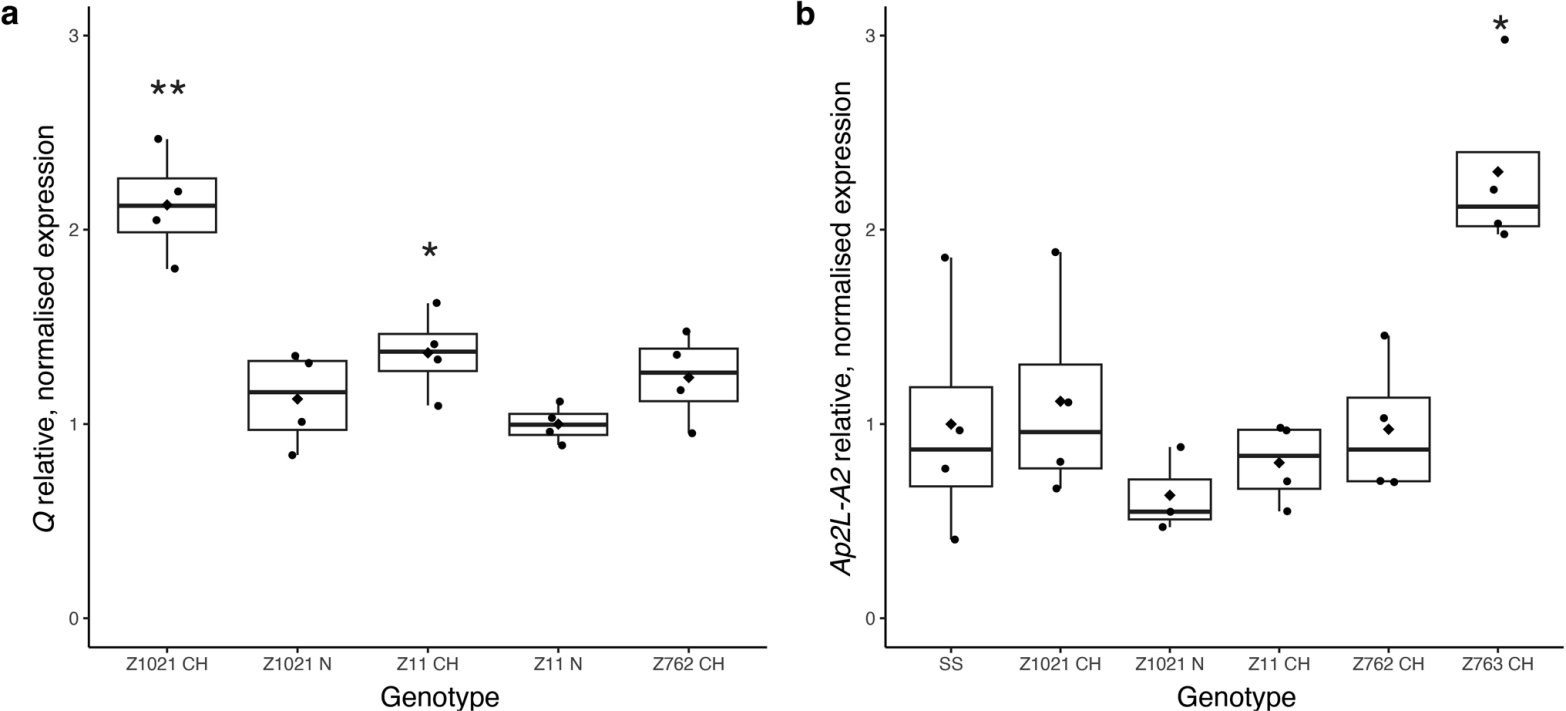
Expression of two members of the *Ap2L* transcription factor family is elevated in Zeb-treated plants with CH phenotype. **a** *Q *expression is elevated in CH plants from Z1021 and to a lesser extent in Z11. Testing by T test (2 tailed, unequal variance) *Q* expression is significantly different between Z1021 CH and Z1021 N and between Z11 CH and Z11 N. Only Z1021 CH showed significantly elevated expression when compared across all samples in an ANOVA (P < 0.05) ** P < 0.001; * P < 0.05. **b** Expression of *Ap2L-A2* is elevated in CH plants from family Z763* P < 0.05 (T test Z763 vs SS as there were no Z763 N plants available)

Elevated expression of *Q*, a major domestication gene located on chromosome arm 5AL, has previously been shown to affect the internode length of both the wheat spike and stem, to be associated with altered floret development and to increase spikelet density similar to the CH phenotype in Z11 and Z1021 (Fig. 2; Table 2; Supplementary Table S2) (Simons et al. 2006; Förster et al. 2012, 2013; Greenwood et al. 2017). As the copy number of chromosome arm 5AL correlated with the severity of the CH phenotype in plants from families Z11 and Z1021, we compared the expression of *Q* in CH and normal (N) apices (Supplementary Fig. S4 & S5) harvested from Z1021 or Z11. In both families, *Q* expression was significantly higher in apices of CH appearance than in the N counterpart (Fig. 5a). In family Z1021, there was a twofold increase in *Q* expression, whereas for family Z11 the difference in *Q* expression between CH and N apices was only 1.5-fold, comparable to that reported in Greenwood et al. (2017). *Q* expression was not increased in apices of either Z762 (Fig. 5a) or Z763 (not shown).

Q is a member of the *Ap2L* transcription factor family in wheat; elevated expression of either the A or B homeoalleles of *Ap2L2*, another member of this family, also results in a CH phenotype (Debernardi et al. 2020). Z763 showed significantly elevated expression of *Ap2L-A2* in the apex (Fig. 5b). The expression of neither *Ap2L-A2, Ap2L-B2* nor *Ap2L-D2* was elevated in CH apices from family Z762 (not shown).

## Discussion

The improvement in existing wheat cultivars through traditional breeding methods requires multiple generations of selection after the initial cross has been made. We have tested the concept that treatment with Zeb, which causes a transient loss of methylation, could give rise to novel, epigenetically based heritable phenotypes in an existing wheat cultivar, bypassing the need to make genetic crosses. As discussed below, while we did observe heritable phenotypes, these generally appeared to be caused by large-scale chromosomal rearrangements rather than discrete changes in methylation of specific genes.

Given that DNA methylation, particularly upstream of the transcription start, is associated with gene silencing, loss of DNA methylation is predicted to cause dominant, gain-of-function mutations. We identified eight treated plants with shortened internodes at the tip of the spike, a phenotype that we termed clubhead (CH). This phenotype was inherited in the progeny of six of these plants and behaved as a dominant trait in all six lines. The appearance of multiple plants with a common phenotype was somewhat surprising because changes in DNA methylation resulting from Zeb treatment would be predicted to occur at random across the genome and to affect only one allele at any locus (Griffith et al. 2016). Surprisingly, two of the Zeb-treated plants appeared to be homozygous for the causal (epi)-mutation leading to the CH phenotype. This could occur through cross-talk between alleles at the affected locus (Chandler and Alleman 2008; Greaves et al. 2012). The molecular basis for the homozygous CH phenotype in these two families (Z762 and Z763) has not been ascertained.

We focussed on two other families, Z11 and Z1021, because some features of the CH phenotype in those families suggested that it could have an epigenetic basis. These features include: (1) the frequency of transmission, which was generally less than a 3:1 segregation predicted for a dominant trait (Tables 2 and 4); (2) the variable severity of the phenotype among the progeny of plants that were judged to be homozygous for the molecular change caused by Zeb treatment (Fig. 2 & 3); (3) complete reversion of the CH phenotype to N spike architecture in some progeny of presumed homozygous plants (Table 4). Some of these features could equally be associated with low transmission of a chromosome with a structural rearrangement, a known consequence of Zeb treatment (Cho et al. 2011; Liu et al. 2015; Ma et al. 2016).

Genomic sequencing of DNA from plants of families Z1021 and Z11 revealed that the CH phenotype in both families was associated with the duplication of part or all of chromosome 5A. In family Z1021, there was a duplication spanning approximately 70% of chromosome 5A, whereas family Z11 had an additional copy of the entire 5A chromosome. These observations were confirmed and extended by a cytogenetic analysis, which revealed an additional copy of 5A (Z11) and the presence of one or more copies of a novel telocentric chromosome comprising the long arm of chromosome 5A (t5AL; Z1021). Genomic sequencing of sibling plants from family Z1021 where the severity of the CH phenotype ranged from normal to extreme dwarf showed that the severity of the phenotype correlated with the copy number of t5AL. Normal plants lacked chromosome t5AL; plants with a mild CH phenotype had one copy of t5AL in addition to two normal copies of 5A whereas plants showing a moderate CH phenotype had two copies of t5AL in addition to two copies of 5A, or three copies of t5AL in addition to a single copy of 5A. Plants with a severe CH phenotype had three copies of t5AL plus two copies of chromosome 5A.

The observation that families Z11 and Z1021 were trisomic for chromosome 5A or part thereof readily explains the observed transmission frequency and reversion of the CH phenotype as additional chromosomes are inherited at variable frequencies, depending on the gamete in question and the structure of the chromosome. At metaphase I of meiosis, the three homologous chromosomes can pair in different trivalent configurations (including linear, frying pan or Y shaped), as a bivalent plus one univalent or, rarely, as three univalents (Talukdar 2013). A plant with 2n + 1 complement of chromosomes would be expected to produce gametes with n and n + 1 chromosomes at equal frequency but this is rarely observed for either male or female gametes (Lindstrom 1936). The extra chromosome is frequently eliminated at meiosis due to lagging of the extra chromosome at anaphase I. Gametes with an n + 1 chromosome composition show reduced viability; in particular, pollen with n + 1 chromosomes has delayed maturation and slow pollen tube growth. In wheat, functional male gametes preferentially have n rather than n + 1 chromosomes as competition strongly favours 21 chromosome pollen, i.e. certation (Sears 1953). It is not clear from our analyses whether the presence of two copies of t5AL allows for more stable segregation of this chromosome, but the formation of quadrivalents with 5A may be associated with irregular disjunction as seen in tetraploid *Datura* (Lindstrom 1936).

Elevated expression of *Q* causes compaction of the spike and dwarfing, with higher expression levels causing a more severe phenotype (Simons et al. 2006; Förster et al. 2012; 2013; Greenwood et al. 2017). Elevated *Q* expression is also associated with abnormal floral development (Greenwood et al. 2017). Similar floral abnormalities were observed in the CH spikes on plants from both families Z11 and Z1021. *Q* expression was compared in apices harvested from plants from families Z11 and Z1021 that had either CH or normal spike morphology. Expression of *Q* was significantly elevated in the CH apices (~ twofold) harvested from family Z1021 when compared with apices from a sibling line that showed normal spike architecture. In Z11, *Q* expression was also elevated (~ 1.5 fold) in CH apices relative to normal apices from the same lineage consistent with the lower copy number of 5AL in this family.

In addition to identifying a partial duplication of chromosome 5A as being the likely cause of the CH phenotype in plants from family Z1021, genomic sequencing showed that the copy number of a 5.7-Mb region of 5A, spanning ~ 252,600,000 bp – 258,300,000 bp, was the same as that seen in SS, independent of the number of copies of t5AL. This region lies within a segment of the chromosome with reduced sequence coverage in SS and, based on mapping data for other wheat cultivars, is probably adjacent to the centromere. This observation suggests that a region of pericentric DNA has been deleted from t5AL but is retained in the two normal copies of chromosome 5A. There has also been a pericentric deletion of approximately 2 Mb on one copy of 5A in family Z11. The deletion in Z11 is almost entirely within the region deleted in Z1021, spanning an interval from approximately 252,500,000 bp to 254,500,000 bp.

Loss of DNA methylation affects chromosome structure, association and somatic homologous recombination in various plant species. Demethylation following 5AzaC treatment of wheat has been shown to cause decondensation of centromeres and to reduce the level of both homologous and non-homologous association in root cells (Vorontosova et al. 2004). Similarly, reduced condensation of centromeres has also been reported in Arabidopsis *ddm1* and *metI* mutants and to a lesser extent in Zeb-treated plants (Soppe et al. 2002; Baubec et al. 2009). Loss of both DNA methylation in non-CG sequences and H3K9me2 is associated with increased double-stranded DNA breaks and crossover formation in pericentric heterochromatin during meiosis (Underwood et al. 2018; Fernandes et al. 2024). Recombination around the centromere during meiosis can cause mis-segregation and aneuploidy in different organisms (Koehler et al. 1996; Lamb et al. 1996). In Zeb-treated plants, aneuploidy most likely arose during a mitotic division because the CH phenotype was observed in plants that had not undergone meiosis. This raises the question of whether loss of these epigenetic marks also affects recombination around the centromere and chromosome segregation during mitosis. It is worth noting that the few reported examples of non-disjunction at Meiosis II were associated with pericentric exchange (Koehler et al. 1996; Lamb et al. 1996).

Zeb treatment increased the frequency of somatic homologous recombination as measured by homologous recombination trap lines in Arabidopsis (Pecinka et al. 2009). In this case, the recombination trap DNA was not methylated and so it was proposed that enhanced recombination may result from DNA damage, which occurs when the cytosine analogue, Zeb, is incorporated into DNA, trapping DNA methyltransferases and thereby blocking the DNA replication machinery (Zhou et al. 2002; Pecinka et al. 2009). Zeb has also been shown to cause DNA strand breakage in cancer cells (Ruiz-Magana et al. 2012). We are unable to determine whether decondensation of pericentric DNA was associated with an unequal recombination between repeated DNA elements or whether DNA damage/(mis)repair led to deletion of pericentric DNA on chromosome 5A in Z11 and Z1021.

Trisomy following Zeb treatment has been previously reported for a rye chromosome in Zeb-treated Triticale (Ma et al. 2016), but the mechanism associated with the chromosome duplication was not determined. As both families in which we observed trisomy of 5A have a pericentric deletion on this chromosome, we suggest that this deletion could be a precipitating factor in the non-disjunction of sister chromatids during mitotic anaphase to give one daughter cell that has three copies of chromosome 5A, with the other being monosomic for 5A.

Breakage of an unpaired chromosome 5A at a subsequent mitotic or meiotic division could have given rise to t5AL in Z1021 or its progeny. Breakage of 5A must have been followed by chromosome repair, which, in wheat, can occur in both the gametophyte and sporophyte. Repair involves the de novo addition of telomeric repeats at the broken chromosome end (Kynast et al. 2000; Friebe et al. 2001); repair is a gradual process and broken chromosomes need to undergo several cycles of mitotic divisions to acquire the full complement of telomeric repeats (Friebe et al. 2001). On rare occasions, we observed sibling plants from Z11 where the severity of the CH phenotype ranged from normal to extreme dwarf (Fig. 2d). In Z1021, varying severity of the phenotype among siblings correlated with the copy number of t5A (Fig. 3). We have not determined whether the entire chromosome 5A is present in increasing copy number to generate the graded series of phenotypes in Z11, or whether it was associated with formation of a telocentric 5A similar to that seen in Z1021.

It has variously been suggested that the demethylating agent Zeb could be used to improve wheat breeding by facilitating the introduction of genes from wild relatives (Cho et al. 2011), for gene and genome studies (Ma et al. 2016) or to activate genes silenced by DNA methylation (this study). The fact that even low concentrations of Zeb can cause major chromosomal aberrations (Cho et al. 2011) that are likely to have unintended consequences suggests that Zeb treatment cannot be used to enhance wheat breeding by reversing transcriptional gene silencing. Treatment with 5 Aza-C has been associated with the appearance of novel phenotypes in both Brassica and strawberries, but it remains to be seen whether these phenotypes are caused by a heritable loss of DNA methylation (Amoah et al. 2012; Xu et al. 2016a, b). Zeb treatment may still be of use to facilitate the introgression of foreign DNA but must be accompanied by additional analyses to identify chromosomal rearrangements.

## Supplementary Information

Below is the link to the electronic supplementary material.

Supplementary file1 (PDF 1066 KB)

Supplementary file2 (DOCX 503 KB)

## Data Availability

Raw sequencing files have been deposited in NCBI SRA under accession PRJNA1152359.
