# Supplementary material for: Testing the potential of zebularine to induce heritable changes in crop growth and development
Source: Theor Appl Genet. 2025 Jan 10;138(1):26. doi: 10.1007/s00122-024-04799-3 (PMC11723894; doi:10.1007/s00122-024-04799-3)
Supplement: Supplementary file 1 — Supplementary file1 (PDF 1066 KB) [file 122_2024_4799_MOESM1_ESM.pdf]

Supplementary Figure 1

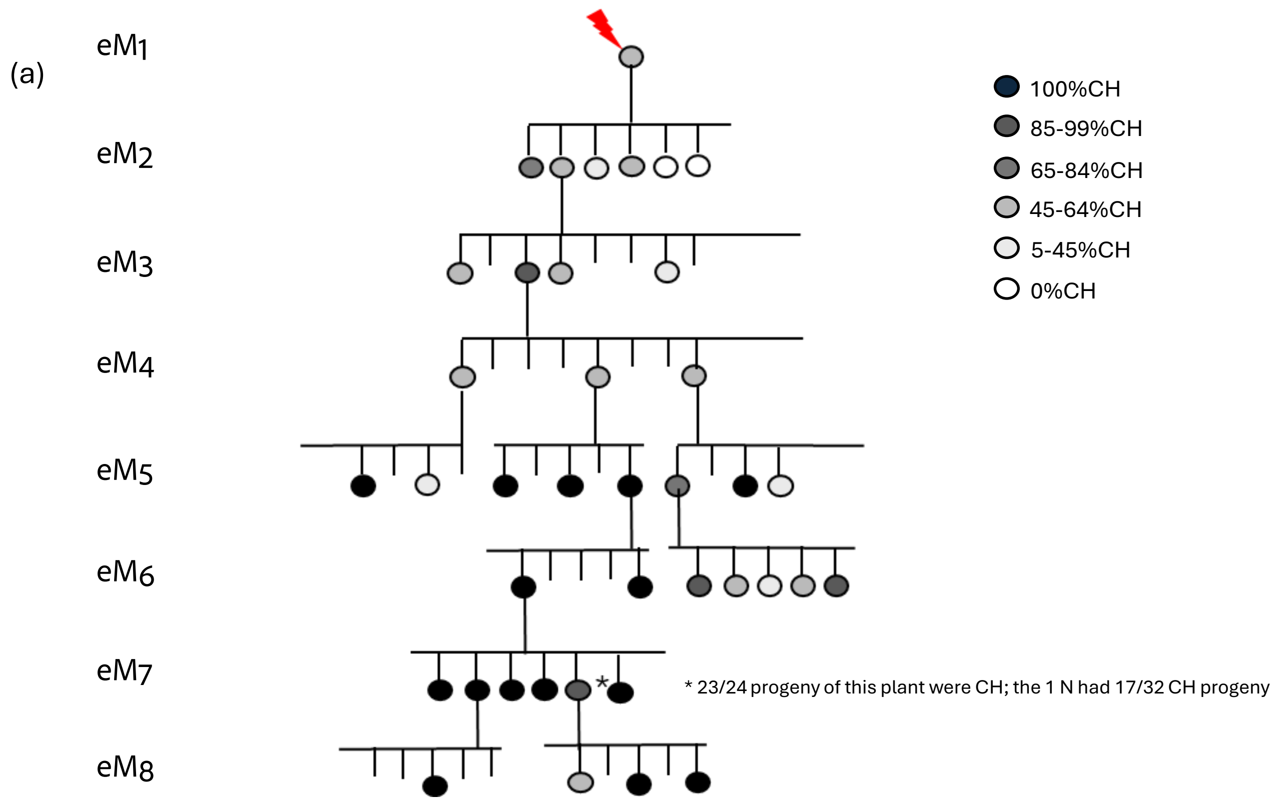

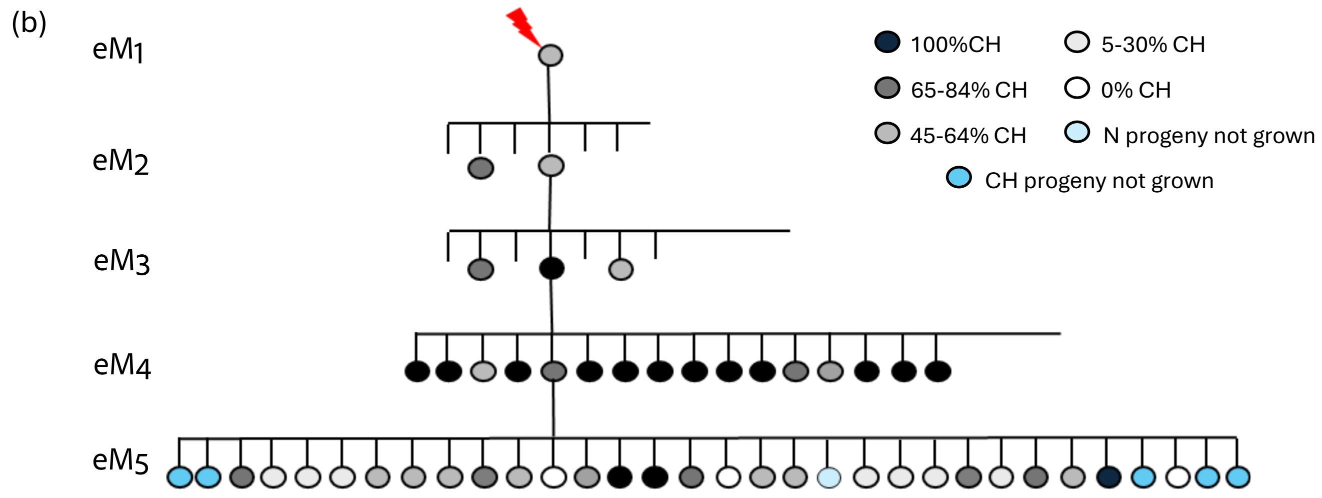

Supplementary Figure 1. Pedigrees of families Z11 and Z1021

(a) A partial pedigree of family Z11 showing the variable transmission of the CH phenotype. The colour of the dots indicates the proportion of progeny that displayed the CH phenotype, rather than the phenotype of the plant

(b) A partial pedigree of family Z1021 showing the variable transmission of the CH phenotype. The colour of the dots indicates the proportion of progeny that displayed the CH phenotype, rather than the phenotype of the plant

Supplementary Figure 2.

(a)

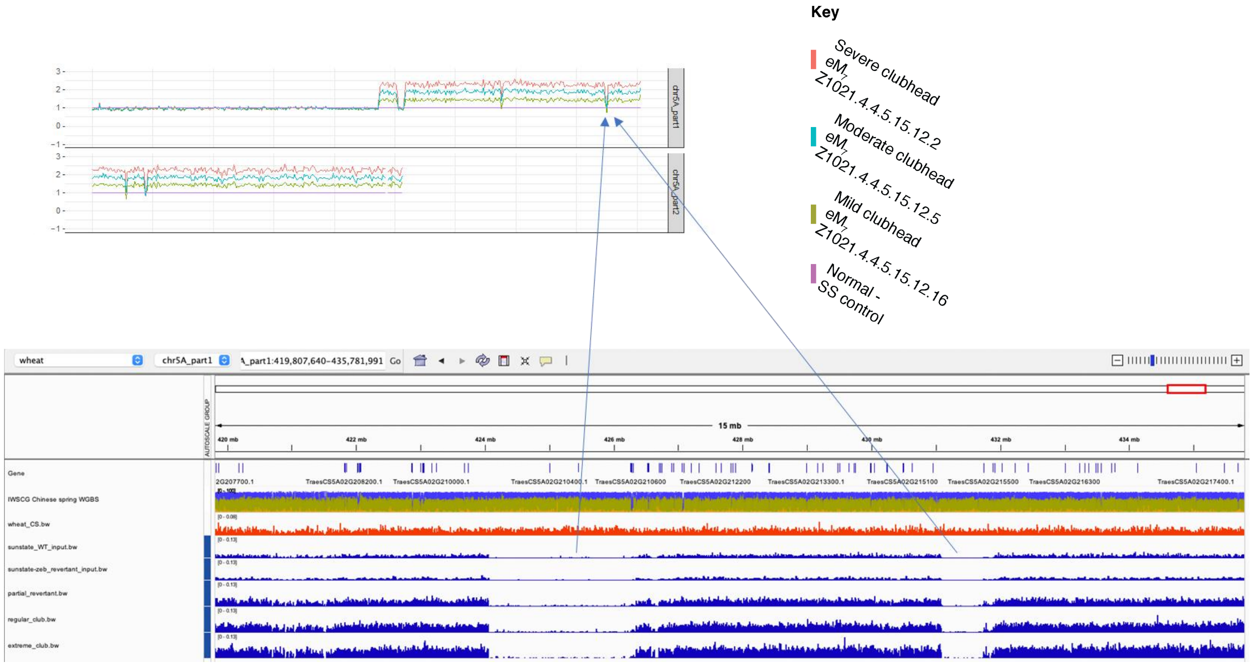

(b)

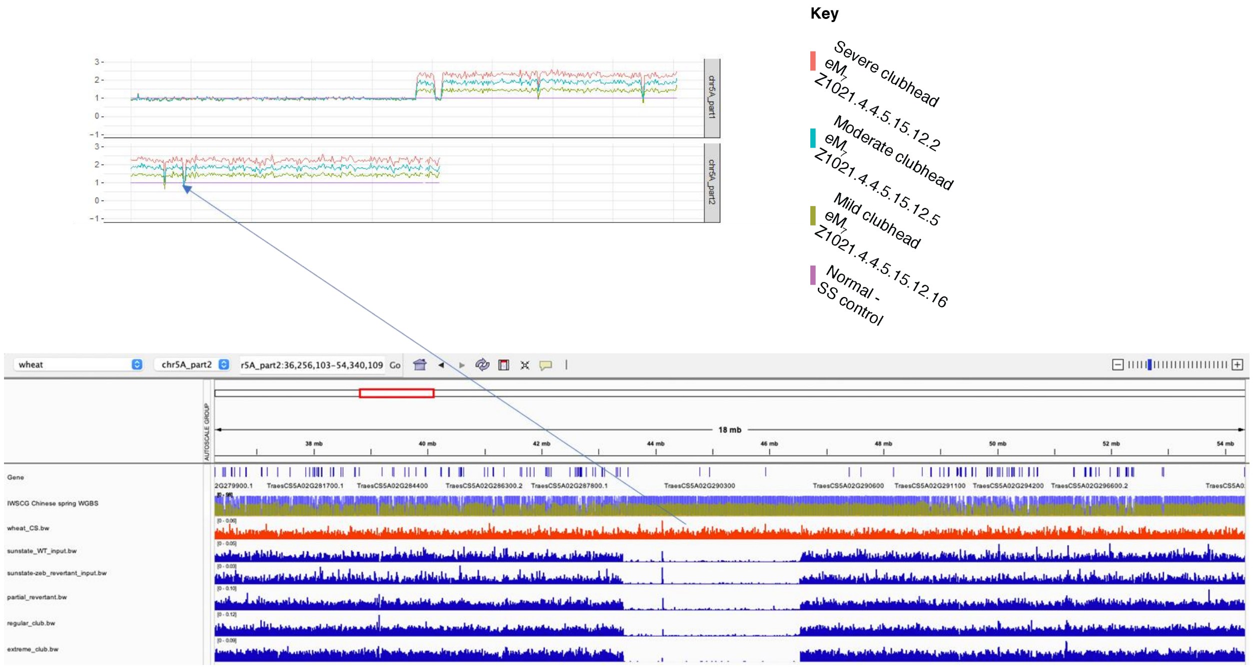

(c)

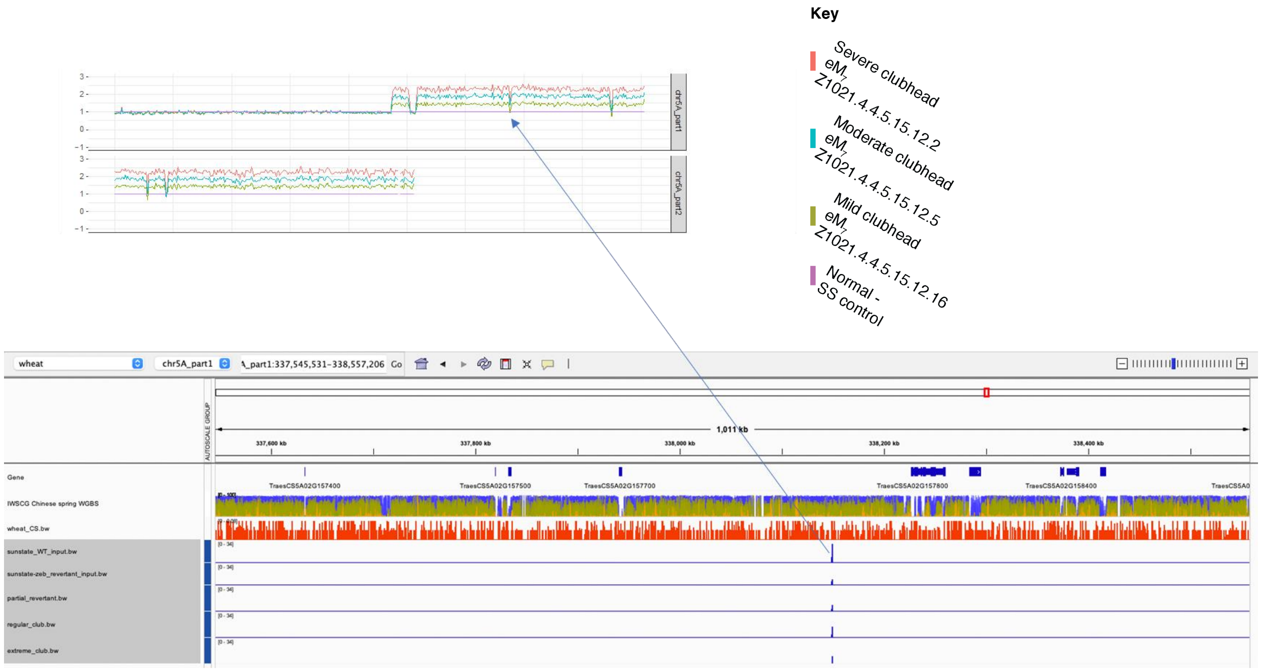

(d)

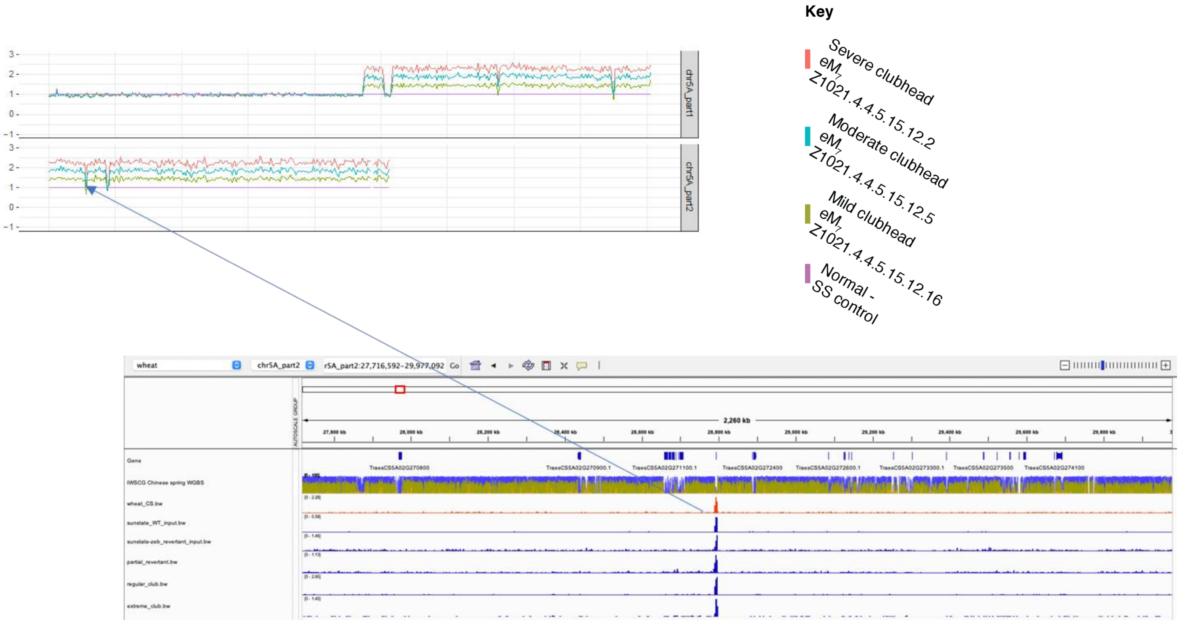

Supplementary Figure 2. Sequencing artefacts identified in sequence traces from t5AL

(a) Artefact due to structural variants in SS bases 424,000,000 – 426,000,000 and 431,000,000 – 432,000,000

(b) Artefact due to structural variants in SS bases 44,000,000 – 46,000,000

(c) Artefact due to abnormally higher coverage in SS at ~ 338,000,000. Maybe Tn5 artefact or due to repetitive sequences

(d) Artefact due to abnormally higher coverage in SS at ~28,000,000. Maybe Tn5 artefact of due to repetitive sequences

Supplementary Figure 3

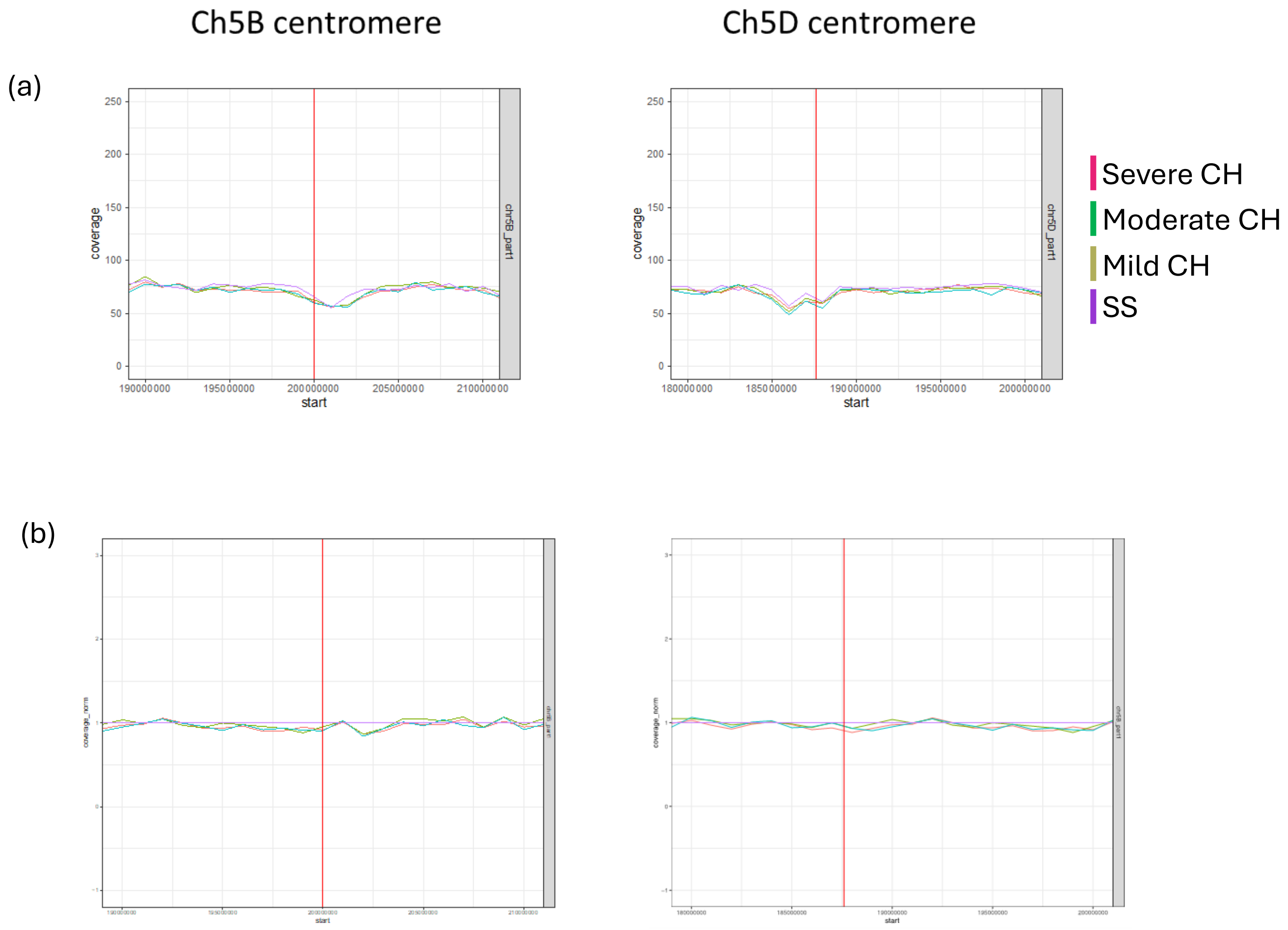

Supplementary Figure 3. Sequence traces for Chromosome 5B and Chromosome 5D for plants from family Z1021

(a) Sequence traces for DNA from plants Z1021.4.4.5.15.12.2, Z1021.4.4.5.15.12.16, Z1021.4.4.5.15.12.5, Z1021.4.4.5.12.2.16 compared to SS. The pericentric region shows reduced coverage in SS and plants from Z1021 relative to CS. The red line marks the estimated start of the centromere in Chinese Spring.

(b) Sequence coverage of chromosome 5B and Chromosome 5D is normalised to SS. There is no evidence for the deletion of pericentric DNA in these chromosomes in plants from family Z1021

Supplementary Figure 4

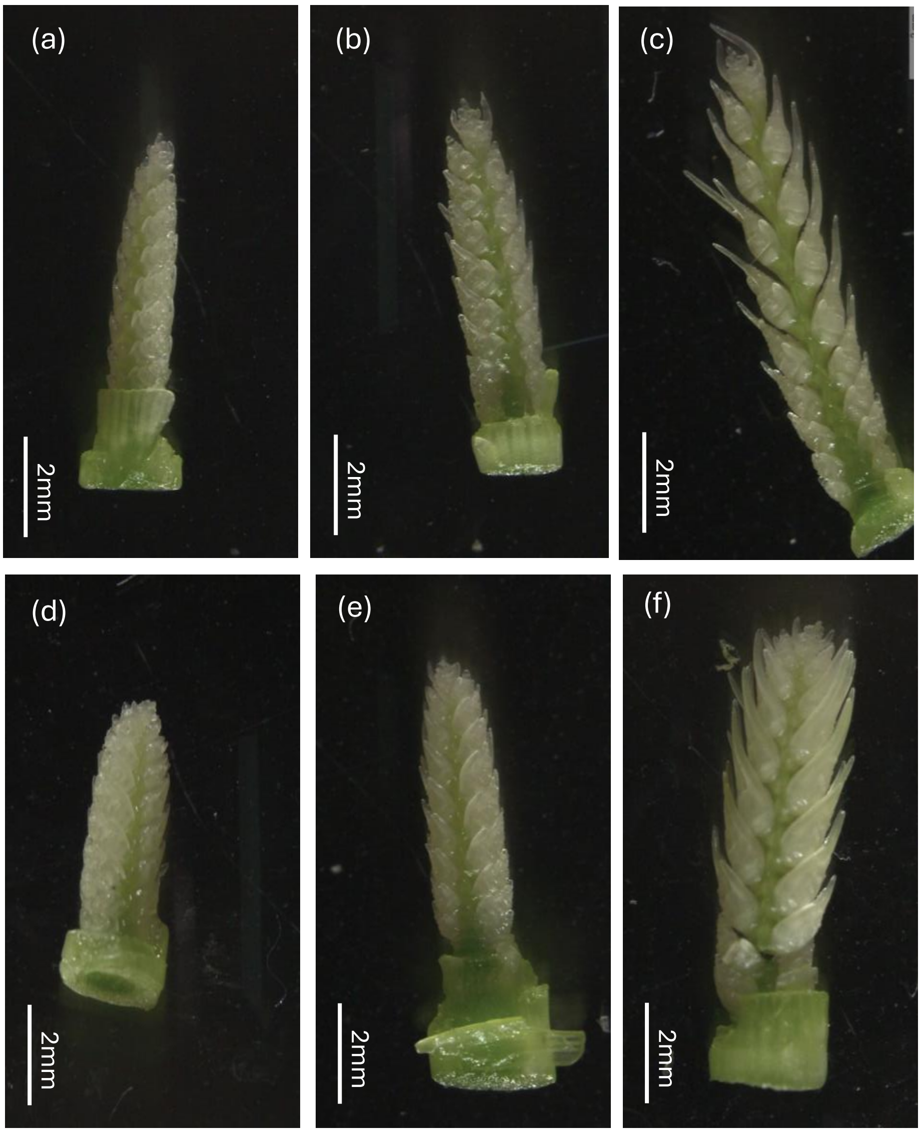

Supplementary Figure 4. A pool of three apices at different developmental stages were used for reverse-transcription qPCR.

(a), (b), (c) Apices from progeny of eM_6 Z1021.4.4.5.17.1, a plant with normal spikes

(d), (e), (f) Apices from progeny of eM_6 Z1021.4.4.5.15.12, a plant with CH spikes

Supplementary Figure 5

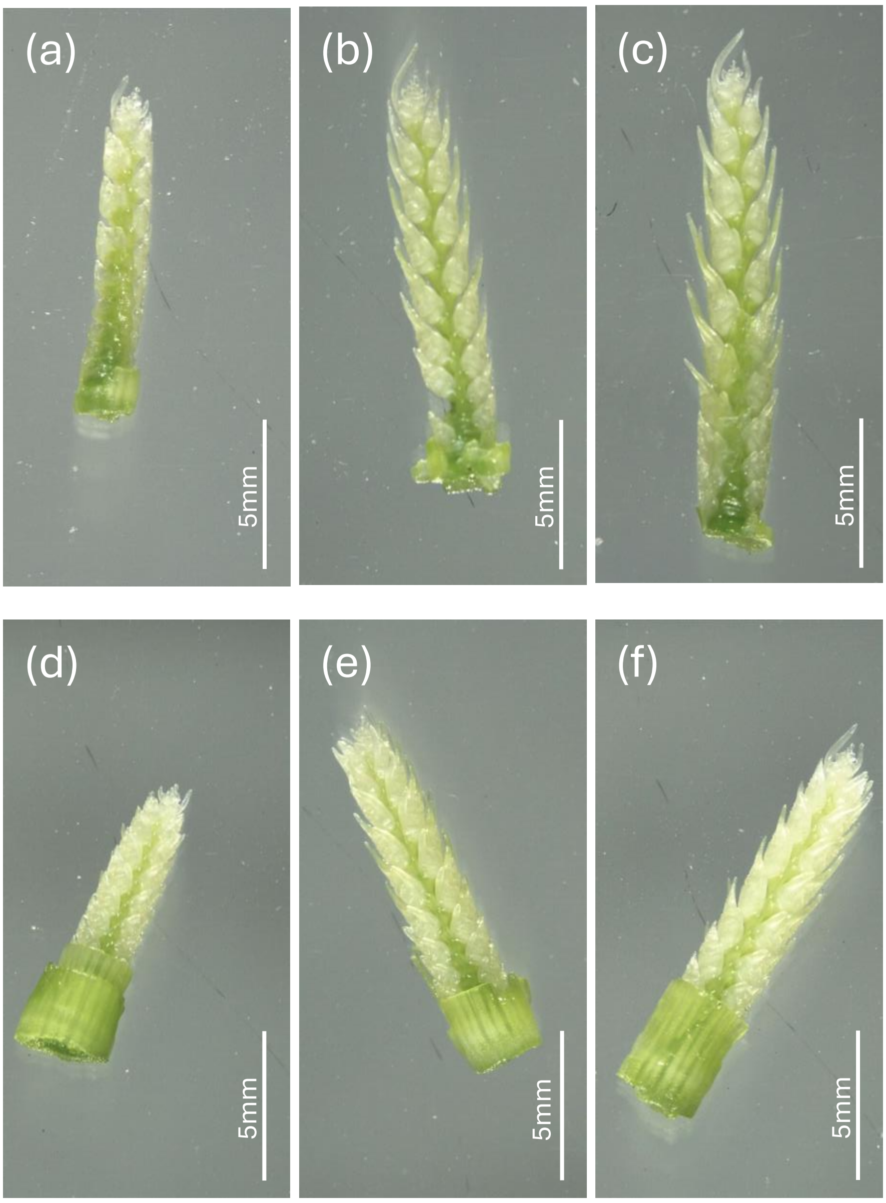

Supplementary Figure 5. A pool of three apices at different developmental stages were used for reverse-transcription qPCR.

(a), (b), (c) Apices from progeny of eM_9 11.2.3.5.21.8.18.5.2, a plant with normal spikes

(d), (e), (f) Apices from progeny of eM_9 Z11.2.3.5.21.8.18.24.6, a plant with CH spikes
