# Supplementary material for: Testing the potential of zebularine to induce heritable changes in crop growth and development
Source: Theor Appl Genet. 2025 Jan 10;138(1):26. doi: 10.1007/s00122-024-04799-3 (PMC11723894; doi:10.1007/s00122-024-04799-3)
Supplement: Supplementary file 2 — Supplementary file2 (DOCX 503 KB) [file 122_2024_4799_MOESM2_ESM.docx]

**Supplementary Table S1.** Whole genome sequence data

| **Supplemental table WGS samples** |  |  |  |  |
| --- | --- | --- | --- | --- |
|  |  |  |  |  |
| **Sample_name** | **Phenotype** | **Lineage** | **Plant_ID** | **Reads** |
| wheat_SS_control_normal_SS_1 | normal | SS | SS | 17714820 |
| wheat_SS_zebularine_normal_z1021_1 | normal | Z1021 | Z1021.4.4.5.12.2.1, Z1021.4.4.5.12.2.7, Z1021.4.4.5.12.2.16 | 17297854 |
| wheat_SS_zebularine_extreme-clubhead_z1021_2 | severe CH | Z1021 | Z1021.4.4.5.15.12.2 | 77474970 |
| wheat_SS_zebularine_mild-clubhead_z1021_3 | mild CH | Z1021 | Z1021.4.4.5.15.12.16 | 57881021 |
| wheat_SS_zebularine_clubhead_z1021_4 | CH | Z1021 | Z1021.4.4.5.15.12.5 | 68956680 |
| wheat_SS_zebularine_normal_z11_1 | normal | Z11 | Z11.2.3.5.8.8 | 58095624 |
| wheat_SS_zebularine_normal_z11_2 | normal | Z11 | Z11.2.3.5.8.10 | 21692508 |
| wheat_SS_zebularine_mild-clubhead_z11_3 | mild CH | Z11 | Z11.2.3.5.3.5 | 26259399 |
| wheat_SS_zebularine_mild-clubhead_z11_4 | mild CH | Z11 | Z11.2.3.5.3.10 | 30584302 |
| wheat_SS_zebularine_clubhead_z11_5 | CH | Z11 | Z11.2.3.1.13.3 | 38989568 |
| wheat_SS_zebularine_clubhead_z11_6 | CH | Z11 | Z11.2.3.1.13.5 | 24848842 |

**Supplementary Table 2.** Spikelet density (number of spikelets/length of spike) was increased CH spikes compared to spikes with N architecture.

| **Phenotype** | **CH** | **N** |
| --- | --- | --- |
|  | 3.8 | 2.5 |
|  | 3.2 | 2.4 |
|  | 3.2 | 2.5 |
|  | 3.2 | 2.3 |
|  | 3 | 2.5 |
|  | 3.3 | 2.3 |
|  | 3.6 | 2.4 |
|  | 3 | 2.3 |
|  | 2.9 | 2.4 |
|  | 3.8 | 2.4 |
|  | 3.4 | 2.4 |
|  | 3.1 |  |
|  | 3.4 |  |
|  | 3.1 |  |
|  | 3.0 |  |
|  | 3.3 |  |
|  | 3.1 |  |
|  | 3.5 |  |
|  | 3 |  |
|  | 3.3 |  |
|  | 3.2 |  |
| **Average1** | **3.3* ± 0.05** | **2.4* ± 0.02** |

*These values are significantly different on a T test (2 tails) p=2.12707E-13

1 mean ± SEM
